# The Safety and Efficacy of Phage Therapy for Bone and Joint Infections: A Systematic Review

**DOI:** 10.3390/antibiotics9110795

**Published:** 2020-11-10

**Authors:** Alex L. Clarke, Steven De Soir, Joshua D. Jones

**Affiliations:** 1 Infection Medicine, Edinburgh Medical School: Biomedical Sciences, University of Edinburgh, Chancellor’s Building, 49 Little France Crescent, Edinburgh EH16 4SB, UK; alexlilyclarke@gmail.com; 2 Laboratory for Molecular and Cellular Technology, Queen Astrid Military Hospital, Rue Bruyn, 1120 Brussels, Belgium; steven.desoir@uclouvain.be; 3 Cellular & Molecular Pharmacology, Louvain Drug Research Institute, Université Catholique de Louvain (UCLouvain), avenue E. Mounier 73, 1200 Brussels, Belgium

**Keywords:** bone infection, osteomyelitis, phage therapy, prosthetic joint infection, systematic review

## Abstract

Bacterial resistance to antibiotics has catalysed interest in alternative antimicrobial strategies. Bacteriophages (phages) are viruses of bacteria with a long history of successful therapeutic use. Phage therapy is a promising antibacterial strategy for infections with a biofilm component, including recalcitrant bone and joint infections, which have significant social, financial and human impacts. Here, we report a systematic review of the safety and efficacy of phage therapy for the treatment of bone and joint infections. Three electronic databases were systematically searched for articles that reported primary data about human phage therapy for bone and joint infections. Two authors independently assessed study eligibility and performed data extraction. Seventeen reports were eligible for inclusion in this review, representing the treatment of 277 patients. A cautionary, crude, efficacy estimate revealed that 93.1% (*n* = 258/277) achieved clinical resolution, 3.3% (*n* = 9/277) had improvement and 3.6% (*n* = 10/277) showed no improvement. Seven of the nine reports that directly commented on the safety of phage therapy did not express safety concerns. The adverse effects reported in the remaining two were not severe and were linked to the presence of contaminating endotoxins and pre-existing liver pathology in a patient treated with high-titre intravenous phage therapy. Three other reports, from 1940–1987, offered general comments on the safety of phage therapy and documented adverse effects consistent with endotoxin co-administration concomitant with the use of raw phage lysates. Together, the reports identified by this review suggest that appropriately purified phages represent a safe and highly efficacious treatment option for complex and intractable bone and joint infections.

## 1. Introduction

In his Nobel Lecture, 17 years after his serendipitous discovery of penicillin in 1928, Alexander Fleming warned that the misuse of penicillin could select for resistant bacteria [1]. Today, almost 100 years on, modern medicine is confronted with the ‘antimicrobial resistance crisis’. This crisis threatens, at great economic and human cost, to undermine substantial progress across medicine and surgery.

Bacteriophages (phages) are viruses of bacteria whose discovery in 1915 and 1917 predates that of antibiotics [2]. The potential use of phages for the treatment of bacterial infections was quickly realised and throughout the early part of the 20th century phage therapy was ‘en vogue’ across Europe, the Americas and Russia [3,4,5]. However, the discovery of antibiotics catalysed the decline of phage therapy in the geopolitical West, where injudicious use, uncertainty about the nature of phages and manufacturing challenges conspired to dispel enthusiasm in phage therapy [6]. Meanwhile, phage therapy remained a frontline antimicrobial strategy across the former Soviet Union, largely in Russia, the Eliava Institute in Georgia and the Hirszfeld Institute in Poland, where thousands of patients have been successfully treated [7,8].

The antimicrobial resistance crisis has driven a renewed global interest in phage therapy as an adjunctive or alternative antimicrobial strategy. Bacterial resistance to antibiotics is often considered to reflect the presence of antibiotic resistance genes, however the ability to survive (tolerate) antibiotics can also be imparted by the formation of biofilms [9]. A biofilm is a bacterial population, often associated with a surface, encapsulated in a heterogenous extracellular matrix [10]. Biofilms impart a variety of tolerance mechanisms to bacteria, often reducing antibiotic efficacy [9]. The population of bacterial cells in a biofilm is physiologically and genotypically diverse, with <0.1% of bacteria becoming persister cells, which are inherently resistant to antibiotics that target replicating bacteria [11].

Biofilms underlie a wide range of infections, including dental, oral, pulmonary and gastrointestinal infections [12]. Biofilms are particularly important in chronic bone and (prosthetic) joint infections [13,14]. Patients with such infections who are candidates for surgery may receive debridement, antibiotics and implant retention (DAIR) or various degrees of surgical revision. Those who are not candidates for surgery receive suppressive long-term antibiotic therapy. The surgical success rate is variable. For example, it has been estimated that the average ability of DAIR to control periprosthetic joint infection is 61.4%, with efficacy ranging from 11.1% to 100% [15]. These infections carry a significant social, human and financial cost. In 2015 in the UK, it was estimated that the average cost to the National Health Service of a patient undergoing revision of an infected knee was £30,011 [16], while it was previously projected that in the United States the annual cost of treatment of prosthetic joint infections would exceed $1.62 billion by 2020 [17].

Arguably, phage therapy has the potential to transform the treatment of these infections. There have been several cases of the compassionate use of phage therapy for the treatment of bone and joint infections over the last few years. Therefore, to support future developments in the field, this systematic review will, without limitation on study design, collate reports of phage therapy for the treatment of bone and joint infections in humans and evaluate the evidence for safety and efficacy.

## 2. Methods

### 2.1. Search Strategy

Three electronic databases were searched, without limits, for articles published up to July 2020: EMBASE (1980–2020), Ovid MEDLINE^®^ Epub Ahead of Print, In-Process & Other Non-Indexed Citations, Ovid MEDLINE^®^ Daily, Ovid MEDLINE and Versions^®^ (1946–2020) and Web of Science. The Web of Science Core Collection Citation Indexes searched were as follows: Science Citation Index Expanded (1900–2020), Book Citation Index–Science (2005–2020) and the Emerging Sources Citation Index (2015–2020). The search was performed using the following terms: (“bacteriophage*” OR “phage*”) AND (“bone” OR “joint” OR “musculoskeletal” OR “MSK” OR “ortho*” OR “osteo*” OR “peri?prosthetic” OR “prosthe*”) AND (“case*” OR “clinic*” OR “patient*” OR “treat*” OR “trial”). In Ovid, these terms were followed by the suffix ‘.mp.’ and they were searched as topics on the Web of Science platform. This systematic strategy was supplemented using reports known to the authors that described the use of phage therapy for a wide variety of conditions but the titles and abstracts of which would not be detected by the specific search terms used [18,19,20,21,22], sources not widely available or indexed online [23,24,25] and relevant papers that became available after the systematic search date [26,27]. A protocol was not published prior to this study.

### 2.2. Study Selection Criteria

All articles were title and abstract screened. Articles were included if they contained primary clinical data about the use of phage therapy in humans to treat any type of bone or joint infection, including cases of skin and soft tissue infection complicated by osteomyelitis and were available in the English language. Secondary literature was excluded unless it reported primary clinical data unavailable from the primary source. There were no restrictions on study date, type or location. There was no limitation on the purity of phage preparation used and studies using raw bacterial lysate were included. Articles reporting the use of phage-derived products (e.g., endolysins) were excluded. Study selection was carried out independently by two authors (ALC, JDJ), with discrepancies resolved by agreement. Deduplication was performed using Endnote (version X8.0.1). Title and abstract screening and subsequent full-text eligibility screening were performed independently by two authors (ALC, JDJ), with discrepancies resolved by agreement. This review was conducted in accordance with the PRISMA (Preferred Reporting Items for Systematic Reviews and Meta-Analyses) guidelines [28] and a PRISMA checklist was completed (Supplementary File S1).

### 2.3. Data Extraction and Critical Appraisal

The following information was extracted from each eligible study: author(s); date of publication; study location; study type; number of relevant reports; condition microbiology; clinical condition and, where possible, patient age(s) and/or previous treatment(s); details of the phage treatment; treatment schedule and route(s), including details of other ongoing therapies (e.g., antibiotics), where reported; treatment efficacy; where possible, the numbers of patients ‘cured’, ‘improved’ or for which there was ‘no response’; comments or data regarding safety and adverse effects. The term ‘cured’ is used in Supplementary File S2 and is defined here as synonymous with clinical resolution of infection. One article recorded six patients as having ‘transient improvement’ [19]. These were defined as treatment failure for the purposes of this review, as ‘transient improvement’ is a nebulous term that does not imply a period of clinical resolution. The extraction of data from each eligible article was performed independently by at least two of the three authors (ALC, JDJ or SDS), with discrepancies resolved by agreement. All eligible studies were independently assessed by two authors (ALC, JDJ) using the appropriate Joanna Briggs Institute critical appraisal checklist [29], with discrepancies resolved by agreement. The influence of publication bias and selective reporting on the cumulative evidence are considered in the discussion. A Fisher’s exact test was performed using an online GraphPad^®^ tool (GraphPad^®^, San Diego, CA, USA) [30].

## 3. Results

Systematic searching yielded 1783 records. After duplicates were removed, 1102 records remained, published between 1933 and 2020. Ten additional records were identified from other sources. Five were reports that described the use of phage therapy for a wide variety of conditions and therefore the titles and abstracts of these records did not contain the specific search terms used [18,19,20,21,22]; three were from grey literature sources known to the authors not to be indexed or available online [23,24,25,31]; and the authors became aware of two further relevant records published after the systematic search date but that were included for completeness [26,27].

**Figure 1:**
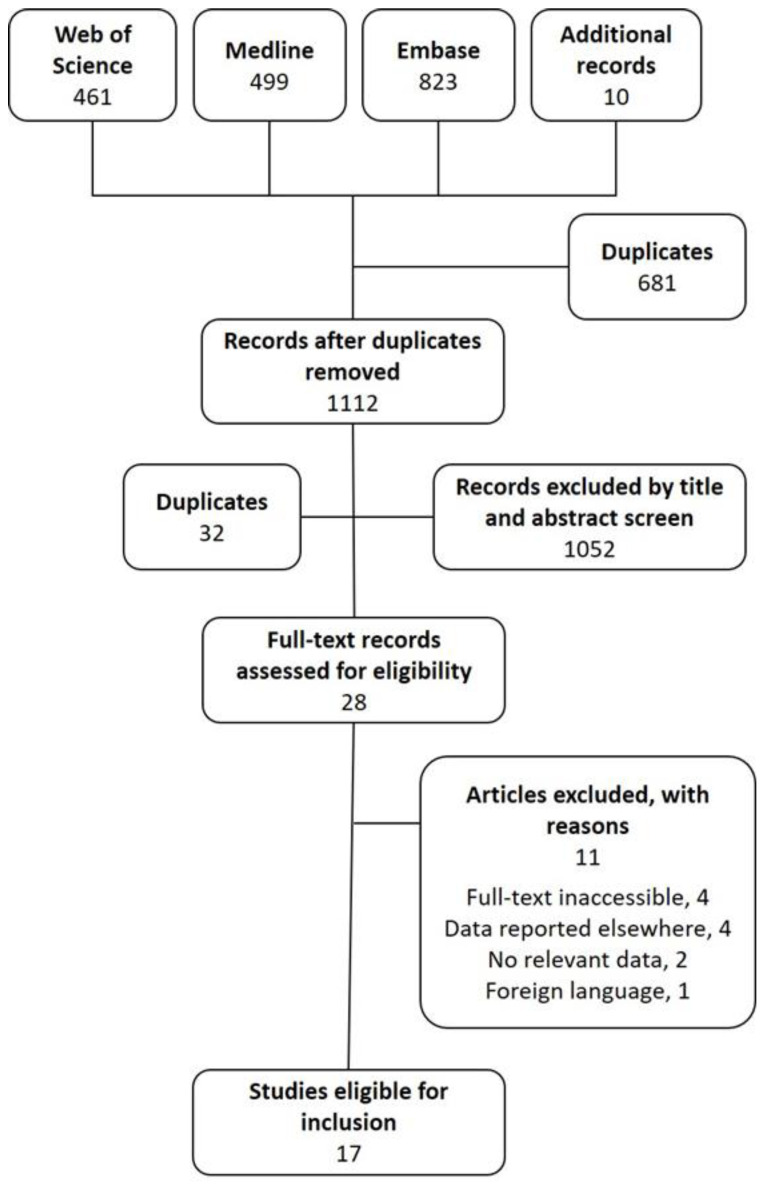
Flow diagram of study selection.

Title and abstract screening revealed a further 32 duplicate records and led to the exclusion of 1052 records that did not meet the inclusion criteria (Figure 1). Of the remaining 28 articles eligible for full-text screening, 11 were excluded because they could not be accessed in full (*n* = 4), contained data reported by a paper already included (*n* = 4), contained no relevant data (*n* = 2) or were not available in English (*n* = 1).

A total of 17 eligible studies were identified for inclusion in this review [19,20,23,24,25,26,27,32,33,34,35,36,37,38,39,40,41]. The data extracted from these studies is shown in Supplementary File S2. These studies were from the Unites States (*n* = 8), France (*n* = 3), Poland (*n* = 1), Belarus (*n* = 1), Belgium (*n* = 1), Germany (*n* = 1), Israel (*n* = 1) and Russia (*n* = 1). There were seven case reports and 10 case series. Critical appraisal highlighted various shortcomings in the quality of reporting but did not provide evidence of bias warranting exclusion (Supplementary File S3).

Together, the 17 records reported data regarding 277 patients treated for osteomyelitis (*n* = 229; 82.7%), post-traumatic bone infections (*n* = 41; 14.8%), prosthetic joint infections (*n* = 5; 1.8%) and septic arthritis/synovitis (*n* = 2; 0.7%). The bacteria most commonly targeted by phage therapy were *Staphylococcus aureus* or other Staphylococcal species (*n* = 9/17) and bacteria from the genera *Streptococcus* (*n* = 3/17) and *Pseudomonas* (*n* = 3/17). Other bacterial genera targeted included *Klebsiella*, *Proteus*, *Acinetobacter*, *Enterococcus* and *Escherichia*. Bacterial resistance to antibiotics was reported in 12 of 17 articles.

Five of seventeen articles reported the use of more than one type of phage to treat patients, reflecting the use of either preformulated or customised cocktails. A further seven articles reported the use of only one type of phage and in five articles details of the phage(s) used were not clear. Screening of bacterial susceptibility to phage therapy was reported by 9 of 17 articles. An additional article reported sensitivity testing for some phages but not others [25], and sensitivity testing by Slopek and colleagues was implied by an earlier, related, publication [42]. Phage therapy was most commonly administered directly into the site of infection during or post surgery (*n* = 6/17), supplemented by intravenous phage in one case. Six of the seventeen reports documented the topical application of phage, supplemented in four cases by oral (*n* = 1) or subcutaneous (*n* = 2) or intramuscular (*n* = 1) injection. Two reports documented subcutaneous phage injection, supplemented by intravenous administration in one case. In two reports, the phage was only administered intravenously. Three reports documented the use of pre-administration neutralization of the gastric mucosa when the phage was administered orally (*n* = 1) or of the wound site when the phage was directly applied (*n* = 2). In the latter cases, 1.4–3.0% sodium bicarbonate was used as a pre-administration wound rinse. One report contained no details about phage administration. The use of antibiotics in combination with phage therapy was reported by 10 of the 17 articles.

A precise efficacy estimate cannot be derived, as co-administered therapies, phages and reporting timepoints and methodology differed between all articles. Notwithstanding these caveats and given that most infections reported were refractory to antibiotics, a crude and cautionary estimate of efficacy can be derived. Together, the 17 articles represented the treatment of 277 bone and joint infection patients of which 93.1% (*n* = 258/277) achieved clinical resolution, 3.3% (*n* = 9/277) had improvement and 3.6% (*n* = 10/277) showed no improvement.

Six of the treatment failures, all classified as ‘transient improvement’ by Slopek and colleagues but classified here as failures (see Methods), were reported among the 11 of 17 articles that reported or implied screening a patient’s bacteria for susceptibility to phage therapy. Together, these 11 reports represented 92.8% (*n* = 257/277) of study patients. Conversely, the six articles that did not report phage sensitivity testing represented 7.2% (*n* = 20/277) of study patients, among which four treatment failures were identified. A Fisher’s exact test comparing the proportions of treatment failure between articles that did or did not report phage susceptibility screening (2.3% vs. 20%) suggested that sensitivity screening reduced the proportion of treatment failures (*p* < 0.05).

Twelve of the seventeen articles commented on safety or adverse effects. Of these, three articles did not comment on aspects of safety specific to the cases included here but commented on the use of the same phage in other groups [19,20,24]. These were classified as indirect safety comments. In 1940, Bernstein reported that 15 of 141 patients showed adverse effects to the subcutaneous injection of phage. Most (*n* = 12/15) had local reactions, such as redness and swelling, and three had generalised reactions (e.g., fever, rigors). In 1963, Baker reported that after the ‘administration of over 35,000 doses’ of raw phage lysate he had observed no allergic reactions and ‘encountered only mild local erythema and swelling and very occasionally a vaccine-type reaction of mild to moderate degree due to overdosage’. Later, in 1987, Slopek and colleagues reported that adverse effects of phage therapy were ‘very rare’, reporting that out of 138 patients two had ‘oral intolerance’ and one displayed ‘allergic symptoms at local application on the wound’. Slopek and colleagues elaborated further, describing the effects of systemic endotoxin release secondary to phage therapy, but reassuringly concluded that phage therapy was ‘safe’, adverse effects were ‘rather rare’, ‘presented no danger for a patient’ and were ‘transient’.

Of the nine articles that directly commented on safety of the cases included in this review, six reported no adverse effects. One article reported that a patient suffered a myocardial infarction and associated complications, but that these were not considered to be related to phage therapy [26]. Of the two remaining articles, Onsea and colleagues (2019) reported that phages were ‘generally well tolerated’ but that one patient experienced local redness and pain. The authors attributed the redness and pain to stowing (extravasation) of the injected phage preparation and an immune reaction to endotoxins present in the phage. Doub and colleagues (2020) reported the treatment of a patient with intravenous and intraarticular anti-Staphylococcal phage therapy. The patient developed non-life threatening transaminitis after the third dose of intravenous phage, which resolved upon cessation of phage therapy. The authors posited that this was secondary to a pre-existing liver condition, which caused a local cytokine response by liver macrophages when exposed to high levels of phage, leading to a local inflammatory response among the patient’s hepatocytes.

## 4. Discussion

This systematic review has collated available English-language publications reporting the use of phage therapy for bone and (prosthetic) joint infections. Although a total of 17 manuscripts were identified, ranging in date from 1933 to 2020 and representing 277 patients, it is likely that this significantly underrepresents the extent to which phage therapy has been used to treat such infections over the last 100 years. Many reports of phage therapy originate from Russia or Eastern Europe, where phage therapy has been used for almost a century [2]. Consequently, there will be manuscripts describing the use of phage therapy for the treatment of bone and joint infections that are unavailable in English and/or not indexed in electronic databases; one such example was identified during this systematic review [43]. As phage therapy is widely accepted in these regions, it is also likely that many routine instances of phage therapy are not deemed to be of publishable value. While these caveats do not detract from the value of collating this evidence, any English-language, predominantly online-based, systematic review is unlikely to offer more than a narrow window on the true extent of evidence regarding the safety and efficacy of phage therapy for any given clinical indication.

The evidence collated by this review suggests that, when used appropriately, phage therapy is highly efficacious for the treatment of bone and joint infections. Approximately 96.4% of the 277 patients included in this review achieved clinical resolution or improvement. Only 10 patients showed no improvement, six of which showed ‘transient improvement’ and four of which were included in a manuscript that did not report phage sensitivity testing or details of the phage therapy used [19,24]. The efficacy of phage therapy is highly dependent upon close matching of the therapeutic phage with the patient’s pathogen. The high proportion of infections that achieved clinical resolution or improvement with phage therapy likely reflects that most studies included in this review (*n* = 11/17) reported the use of phage sensitivity testing, representing 92.8% of patients (*n* = 257/277). However, it is noteworthy that, despite a strong correlation between in vitro and in vivo efficacy [44,45], negative in vitro susceptibility does not always preclude in vivo efficacy [46]. As demonstrated by Nir Paz and colleagues [36], phage therapy has significant potential in the treatment of polymicrobial infections, where a cocktail of phages targeting the various bacteria responsible can be used [47]. Although bacterial resistance to antibiotics was reported in 12 of 17 articles, patients in 10 of 17 articles were treated with phage therapy alongside antibiotics, in some cases despite antibiotic resistance. The use of phage therapy alongside antibiotics reflects the urgent clinical need of many of these patients but, without in vitro efficacy data, may hamper interpretation of the relative in vivo efficacy of each antimicrobial. Notably, there is evidence that antibiotics and phage therapy can be synergistic in some cases, including when antibiotics are used at sub-inhibitory concentrations, which may have broader implications for antibiotic resistance in general [48,49,50]. Importantly, phage therapy acts independent of antibiotic resistance and some phages also possess limited enzymatic anti-biofilm activity, degrading the biofilm extracellular matrix; although complete biofilm clearance may be unlikely [51,52]. Resistance to phage therapy can occur, however this is mitigated by the use of phage cocktails [47]. Combining phage therapy with other anti-biofilm strategies, such as antibiotics or debridement surgery, can enhance biofilm clearance [51,53]. The anti-biofilm activity of some phages makes them particularly suited to the treatment of biofilm-containing chronic bone and joint infections [40,54,55].

The articles included in this review suggest that the use of phages to treat bone and joint infections is safe and without risk of significant adverse effects. Twelve of the seventeen articles commented on safety or adverse effects, with nine commenting directly on the patients in question and three commenting more broadly on safety. Six of the nine articles that directly commented on safety or adverse effects reported no side-effects, with a seventh article highlighting that a patient’s later myocardial infarction was not considered to be related to phage therapy [26]. Of the remaining two articles, Doub and colleagues suggested that their patient’s pre-existing liver pathology explained their observation of non-life threatening reversible transaminitis in response to intravenous phage used at a titre of 2.7 × 10^9^ plaque forming units (PFU)/mL [32]. This would be consistent with the absence of transaminitis among other patients treated with intravenous phage of a similar titre [27,36,56,57]. This suggests that liver function should be closely monitored in patients with pre-existing liver pathology receiving intravenous phage, but that the risk of adverse effects from intravenous phage remains low. Onsea and colleagues’ 2019 report of localised pain and swelling was considered to represent the extravasation of phage and the effect of endotoxins present in the phage preparation [37]. It is known that the administration of endotoxin can cause localised and/or inflammatory responses [58]. Many early reports of phage therapy used raw phage lysates (i.e., a mixture of dead bacteria, phage particles and bacterial culture broth). Such phage lysates often contained high levels of contaminating endotoxin and consequently adverse effects consistent with co-administered endotoxin were observed. When considering comments on safety and adverse effects, it is therefore important to evaluate any available information about the endotoxin content of the phage preparation. For example, the co-administration of endotoxin with raw, impure, phage lysates readily explains the comments of Bernstein, Baker and Slopek, which offered indirect appraisals of safety and documented localised and systemic reactions [19,20,24]. Broadly, the safety of phage therapy identified by the studies included in this review illustrates how advantageous phage therapy is relative to antibiotic therapy, which often causes adverse effects. However, the safety of every different phage used for therapy must be assessed. Extensive literature about the characterisation of phages for therapy is available elsewhere [59,60,61], with primary concerns being the exclusion of phages with lysogenic, toxin or antimicrobial resistance genes. Lastly, five articles did not comment on safety or efficacy, potentially reflecting the inherent tendency of scientific literature to only report ‘significant’ findings. It is important that all future clinical applications of phage therapy comment on safety and adverse effects, even if none were present, and that information about the purity of the phage preparation used is included to inform analysis of any adverse effects.

As mentioned above, this review was limited by being restricted to the English language and predominantly online sources. The systematic review approach is also inherently limited by whether relevant studies are available, accessible or appropriately indexed in the databases searched. Moreover, there is a risk that relevant papers may not be retrieved by systematic searching if they lack the search terms in the fields searched. The reproducible and methodological nature of this systematic review also limited the inclusion of sources of which only an abstract was available. There were three such sources; one was available in full but not in English. From Russia in 2014, Shusharin and colleagues reported the use of intraarticular ‘polyvalent bacteriophage’ to treat 15 patients with gout (*n* = 5) or rheumatoid arthritis (*n* = 10) and associated synovitis of the knee or hip [62]. For 7 of 15 patients, ‘the process was completely arrested’ after two injections. For the remaining 8 of 15 patients, a total of 3–4 injections was required. It was observed that phage ‘has a strong analgesic and anti-inflammatory effect’, ‘pain in the joint passes after 3–24 h after injection’ and that there was no ‘exacerbation of primary disease’. From Romania in 2016, Negut and colleagues reported the use of oral and/or topical phage preparations from the Eliava Institute (Tbilisi, Georgia) in two patients with chronic osteomyelitis [63]. These osteomyelitis patients were treated alongside four other patients with recalcitrant soft tissue infections. The available abstract did not clearly report the outcomes but did state that ‘for all six cases the combined therapy proved to be safe, with no adverse reactions and no adverse changes in laboratory parameters’. From Russia in 2018, a case report by Efremov and colleagues detailed a two-stage reosteosynthesis procedure in which cement containing vancomycin and polyvalent bacteriophage was successfully used to treat a patient with chronic osteomyelitis [43]. Although conference abstracts were not included in the systematic review strategy, an additional conference abstract was identified during this review. From France in 2018, Dublanchet and Patey reported the results of a cohort of 10 patients treated since 2008 [64]. The ‘initial bacteria were eradicated’ but replaced by a different bacterial species in two cases. Although not available in full, these abstracts further underscore the potential of phage therapy as a safe and efficacious treatment option for complicated orthopaedic infections.

Bone and joint infections are often chronic, complex and may often be refractory to antibiotic therapy because of antibiotic resistance and/or biofilm-based tolerance. This systematic review presents English-language data which offers a reassuring evidence base supporting the safety and efficacy of phage therapy for the treatment of these infections. Phage therapy has a long history in the treatment of bone and joint infections and has been successfully used in both ‘cocktail’ and ‘personalised’ formulations. Amid the growing antimicrobial resistance crisis, phage therapy is likely to offer a valuable adjunct or alternative future therapeutic option, especially in clinical indications where biofilm-based antibiotic tolerance occurs.

## Data Availability

Data sharing is not applicable to this article, as no datasets were generated or analysed during the current study.

## Supplementary Materials

The following are available online at https://www.mdpi.com/2079-6382/9/11/795/s1: Supplementary File S1. PRISMA checklist. Supplementary File S2. Phage therapy for the treatment of bone and joint infections, *n* = 17. Supplementary File S3. Critical appraisal.

## Abbreviations

ALT	alanine aminotransferase
AST	aspartate aminotransferase
CRP	C-reactive protein
DAIR	debridement, antibiotics and implant retention
IV	intravenous
MRSA	methicillin-resistant *Staphylococcus aureus*
PFU	plaque forming unit
PRISMA	Preferred Reporting Items for Systematic Reviews and Meta-Analyses
SC	subcutaneous
SPL	*Staphylococcus* phage lysate
WBC	white blood cell
