# Supplementary material for: The Safety and Efficacy of Phage Therapy for Bone and Joint Infections: A Systematic Review
Source: Antibiotics (Basel). 2020 Nov 10;9(11):795. doi: 10.3390/antibiotics9110795 (PMC7697170; doi:10.3390/antibiotics9110795)
Supplement: Supplementary file 1 [file antibiotics-09-00795-s001.zip › Supplementary File S2.docx]

| **Report details** | | **Clinical details** | | | | **Efficacy** | | | | **Safety & adverse effects** |
| --- | --- | --- | --- | --- | --- | --- | --- | --- | --- | --- |
| **Author (year), [citation], location, study type** | **No. of relevant reports and microbiology** | **Condition details** | **Phage sensitivity** | **Phage treatment** | **Treatment schedule and route(s)** | **Outcome** | **Cured** | **Improved** | **No response** |  |
| Albee (1933), [41]  USA  Case series | 100/100  *Staphylococcus* spp.,  *Streptococcus* spp.,  *Mycobacterium tuberculosis,*  *Clostridium perfringens* | Osteomyelitis. | Phage sensitivity testing performed; no data shown. | ‘Laboratory-bred phage’. Target host(s) not reported. | Wound was packed with a vaselin/paraffin mixture with an indwelling catheter through which 10cc of phage were injected once or twice a week for 8 weeks. The process was repeated as needed until complete skin healing was achieved, on average three times (24 weeks). | Average healing time 6 months. | 100 | 0 | 0 | No comment. |
| Bernstein (1940), [24]  Belarus  Case series | 7/25  No microbiological details. | Osteomyelitis. | No phage sensitivity testing reported. | No details reported. | Phages were administered by subcutaneous (SC) injection ‘no more than 3 times in increasing doses (1ml, 2ml, 3ml) at intervals of 3 days)’. This was supplemented by direct spraying of phages onto the wound at each dressing change. | 3/7 successfully treated;  4/7 unaffected. | 3 | 0 | 4 | No comment specific to these cases. However, in reference to a separate cohort of 141 patients covered in the same report and treated with phage in the same way the author states: 15/141 showed side effects after SC injection. 12/15 were local reactions (redness, swelling) and 3/15 were generalized reactions (fever, rigors). |
| Baker (1963), [20]  USA  Case series | 1/8  *Staphylococcus aureus* | A 15-year-old male presented with septic synovitis of the left knee and septicaemia caused by *S. aureus*. Condition worsened by day 9 with evidence of a reaction to penicillin. | No phage sensitivity testing reported. | *Staphylococcus* phage lysate (SPL) containing 2 x 10^9^ lysed *S. aureus* and  >10^10^ ‘Gratia’ *Staphylococcus* bacteriophage  per cc. | On day 10 the patient was started on intravenous (IV) SPL once every 4h, supplemented by subcutaneous administration. IV SPL was stopped on day 14. Subcutaneous phage was continued twice daily until day 20 after which it was reduced to 0.5cc once weekly for 6 weeks and then twice monthly for five months. | Marked improvement in 48 hours. An x-ray on day 23 showed no further worsening. The patient was discharged on day 27 with follow-up x-rays at 2 and 4 months both negative. No recurrence was observed in 16 months. | 1 | 0 | 0 | No comments specific to this case. However, the author notes that ‘no allergic reaction to SPL has been authentically reported’ and SPL is ‘non-toxic, non-sensitising and non-irritating’. ‘Administration of over 35,000 doses of SPL […] over the past 12 years has confirmed the safety […] I have encountered only minimal local erythema and swelling and very occasionally a vaccine-type reaction of a mild to moderate degree due to overdoseage’. |
| Matusis (1974), [25]  Russia  Case series | 65/89*  *Report states 94 patients but data for 5 are absent.  93% of cases were Staphylococcal infections. | Osteomyelitis. | 72% of isolated bacteria were susceptible to the corresponding phage (produced by the Eliava Institute, Georgia). | No details reported. | 12 patients were treated using antibiotic and topical phage therapy.  53 patients were treated using phage applications and/or intramuscular injections. | Among the 12 treated with antibiotics and phage: 100% healing in 0.5-3 months, consolidation in 4-9 months, 2 patients later relapsed.  Among the 53 treated with phage alone: 100% healing in 0.3-2 months, consolidation in 3-6 months (7-9 months in 6 cases), no relapses. | 65 | 0 | 0 | No comment. |
| Slopek *et al.*  (1987), [19]  Poland  Case series | 81/550  *Staphylococcus* spp.,  *Escherichia* spp.,  *Proteus* spp.,  *Pseudomonas* spp.,  *Klebsiella* spp. | Osteomyelitis of long bones (n = 40).  38/40 cases antibiotic resistant.  11/40 polymicrobial. | No phage sensitivity testing reported. However, according to (Slopek *et al.*1983 [42]), sensitivity confirmed, results not shown. | No details. However, according to (Slopek *et al.*1983 [42]), a library of 259 phages was available for use. Crude phage lysates were used therapeutically. | 10ml of phage was given orally 3 times daily, before a meal and after gastric neutralisation. Phages were also applied locally. | The suppurative process was eliminated in 38 cases. Three of which were classed as ‘marked improvement’. Transient improvement was observed in 2 cases. | 35 | 3 | 2 | No comments specific to these patients.  However, according to (Slopek *et al.* 1983 [42]): ‘side effects in the course of phage therapy are very rare. Out of 138 [patients] only 3 cases were recorded of which 2 displayed drug intolerance at oral administration and 1 allergic symptoms at local application on the wound.’  ‘On day 3-5 of phage therapy, hepatalgia  occurred which lasted several hours. This can be accounted for mass liberation of endotoxins resulting from phage  effect on bacteria. [In] severe cases with sepsis, an increase of temperature occurred on day 7 - 8 of phage administration which lasted 24 h.’  ‘Bacteriophages are safe, side effects are rather rare and present no danger for a patient, they are transient and easy for restraint.’ |
|  |  | Post-fracture osteitis of long bones  (n = 41).  37/41 cases antibiotic resistant. 17/41 polymicrobial. |  |  |  | The suppurative process was eliminated in 37 cases. Five of which were classed as ‘marked improvement’. Transient improvement was observed in 4 cases. | 32 | 5 | 4 |  |
| Southwest Regional Wound care Centre (2006), [23]  USA  Case series | 4/28  One patient infected with each of *Streptococcus viridans* or  *Staphylococcus epidermidis.*  No microbiological data for two patients. | Osteomyelitis of the foot/toe.  Co-morbidities were described for 2/4 and were gout (n = 1) and diabetes (n = 1). | No phage sensitivity testing reported. | No details given. | No details of phage therapy provided. Phage was used as part of a varied package of care including debridement, wound care and ‘biofilm management’. | Wounds healed in between ≤10 (n = 3) and 20 (n = 1) weeks. | 4 | 0 | 0 | No comment. |
| Fish *et al.* (2016), [33]  USA  Case series | 5/9  Methicillin susceptible *Staphylococcus aureus* (n= 4);  MRSA (n = 1). | Diabetic toe infection complicated by osteomyelitis. All patients had vascular insufficiency and had failed to respond adequately to antibiotic therapy. | No phage sensitivity testing reported. | Monovalent suspension of anti-staphylococcal phage Sb-1 at ~10^7^-10^8^ PFU/ml (Eliava Institute, Tbilisi). | Once weekly applications of 0.1-0.5cc. Phage was dripped into the wound cavity, which was packed with phage-soaked gauze, covered with petroleum gauze and dry gauze. The phage dressing was left in place for 48h. Phage therapy supplemented standard wound care. | All infections responded and healed in an average of 7 weeks. | 5 | 0 | 0 | No adverse effects observed. |
| Ferry *et al.* (2018), [39]  France  Case report | 1/1  *Pseudomonas aeruginosa*, sensitive only to polymyxins and ceftolozane/ tazobactam. | Postoperative sacro-iliac joint infection in a male patient treated for metastases from disseminated non-small cell lung cancer. | Sensitivity confirmed | A customised cocktail of four anti-*Pseudomonas* phages (Pherecydes Pharma, France), containing each of the phages at 1.2-9.7 x10^8^ ‘cfu/ml’ [presumed PFU/ml] in a final volume of 30ml of 0.9% saline. | During surgical debridement 10 ml of the phage cocktail was introduced into the cavity. The cavity was then filled with compresses soaked with 20ml of phage cocktail before a waterproof dressing was applied. The patient was kept in a ventral decubitus position for 4h to maintain phage contact with the infection site. Phage administration was performed every three days, for a total of four administrations. The patient also received 1200mg colistin locally in 12 cycles per day and intravenous ceftolozane/ tazobactam at 8h intervals. | Surgical reconstruction was undertaken on day 14 after debridement by which time ‘the macroscopic aspect was extremely favourable’. Cultures taken during surgery were negative. | 1 | 0 | 0 | No comment. |
| Ferry *et al.* (2018), [40]  France  Case report | 1/1  Multi-drug resistant *Pseudomonas aeruginosa* and methicillin susceptible *Staphylococcus aureus.* | 80-year-old obese female with type 2 diabetes presented with a four year history of relapsing prosthetic joint infection of the right hip. | Sensitivity of *Pseudomonas* to phage was confirmed. The sensitivity of the *Staphylococcus* was tested retrospectively and 2/3 phages were effective. | Two three-phage cocktails containing 10^9^ PFU/ml of each phage suspended in Dulbecco’s phosphate buffered saline were prepared, one against each of *Pseudomonas* and *Staphylococcus* (Pherecydes Pharma, France). | Both 10ml cocktails were injected into joint before surgical wound closure. Operative cultures grew MSSA, *Enterococcus* and *S. lugdunensis*. The patient was also treated with 850mg/day daptomycin for 3 months and oral amoxicillin (6g/day) and clindamycin (1.8g/day) to month six post-op. During follow-up the patient required a further debridement, antibiotics and implant retention (‘DAIR’) procedure for a *Citrobacter* infection. | 18 months after phage injection the outcome was favourable with no clinical signs of persistent infection. | 1 | 0 | 0 | ‘This salvage treatment was safe’. |
| Fish *et al.* (2018), [34]  USA  Case report | 1/1  Methicillin susceptible *Staphylococcus aureus.* | Diabetic foot infection complicated by osteomyelitis. | No phage sensitivity testing reported. | Monovalent suspension of Staphylococcal phage Sb-1 (Eliava Institute, Tbilisi), titre not reported. | Injections of 0.7cc of phage around the wound site once weekly for 7 weeks. Levofloxacin was administered after the first 7 days of treatment and was stopped after 7 days because of no notable clinical response. No further antibiotics were given. | Complete cure in 7 weeks. Osteomyelitis remained resolved three years later. | 1 | 0 | 0 | No comment. |
| Fish *et al.* (2018), [35]  USA  Case series | 2/6  (4 cases already presented in Fish *et al.*2016).  All cases were *S. aureus* positive with ‘perhaps a second or third organism’. | Chronic non-healing wound. A 27-year-old female with osteomyelitis on the left great toe refractory to 8 weeks of intravenous and 2 weeks of oral antibiotics. | No phage sensitivity testing reported. | Monovalent suspension of Staphylococcal phage Sb-1 (Eliava Institute, Tbilisi), titre not reported. | Once weekly applications of 0.1-0.5cc. Phage was dripped into the wound cavity, which was packed with phage-soaked gauze, covered with Xeroform® gauze and dry gauze. The phage dressing was left in place for 48h. This patient received 3 applications in 2 weeks before no longer being able to receive treatment because of caring for a relative. | Reduced inflammation was apparent 14 days after starting phage; the patient did not return to clinic. In June the patient sent a photo showing the wound had healed. Healing was maintained at an unrelated appointment 2.5 years later. | 2 | 0 | 0 | ‘No adverse effects, tissue breakdown or recurrence of infection were seen, and the progression to closure was smooth and continuous after initiation of bacteriophage therapy.’ |
|  |  | A 71-year-old female with a diabetic toe ulcer. The patient had a *Clostridium difficile* infection secondary to lengthy use of antibiotics and was on vancomycin at the time of presentation for phage therapy. Bone culture revealed *S. epidermidis* and *S. lugdenensis*. |  |  | Following debridement, 0.5cc of phage was injected into the distal toe on three occasions: days 1, 7 and 28. | The ulcer resolved in 8 weeks and there was no recurrence at an unrelated appointment approximately 4 months later. |  |  |  |  |
| Nir-Paz *et al.* (2019), [36]  Israel  Case report | 1/1  *Acinetobacter baumannii* and  *Klebsiella pneumoniae* co-infection. | A 42-year-old male with a trauma-related osteomyelitis of the left tibia. Treatment with serial irrigations and debridements. Neither a six-week course of piperacillin/ tazobactam nor an 8-week course of meropenem and high-dose colistin were sufficient to clear the infection. | Sensitivity confirmed. | Phages against *A. baumannii* (AbKT21φ3) and *K. pneumoniae* (KpKT21φ1) were obtained from the US Naval Medical Research Centre phage bank. | 1ml of each phage (titres not reported) was administered intravenously over 35 minutes for 5 days. The patient was also treated with intravenous meropenem and colistin. *A. baumannii* was still isolated from the wound after 5 days and a second course of AbKT21φ3 was given for 6 days in the same way one week later. | Signs of wound resolution and reduced pain appeared within days. The wound went on to close and associated pain disappeared. No positive cultures were found at follow-up 8 months later. | 1 | 0 | 0 | ‘No deleterious effects of phage therapy were observed during or after the treatments’. |
| Onsea *et al.* (2019), [37]  Belgium  Case series | 4/4  *Staphylococcus epidermidis* and *Pseudomonas aeruginosa* (n = 2); *Staphylococcus aureus* and  *Streptococcus agalactiae* (n = 1);  *Enterococcus faecalis* (n = 1). | Chronic osteomyelitis of the femur (n = 3) or pelvis (n = 1). All patients had previously failed antibiotic therapy. | Sensitivity confirmed. | BFC1 cocktail (Queen Astrid Military Hospital, Brussels). Contains phages against *S. aureus* (ISP) and *P. aeruginosa* (PNM, 14-1). Phages at 10^7^ PFU/ml in 0.9% saline.  PYO phage cocktail (Eliava Institute, Tbilisi). Contains phages against *Streptococcus* spp., *Staphylococcus* spp., *Proteus* spp., *Escherichia* *coli*, *P*. *aeruginosa* and *Enterococcus* spp. | Following debridement, wound drains were placed. The wounds were rinsed with 1.4% sodium bicarbonate followed by 10-40ml of phage solution. A contact time of 10 minutes was allowed. Before wound closure a gentamicin-impregnated sponge soaked in phage solution was placed on the infected bone. Phage solution was applied via the wound drain 3 times daily for 7-10 days. All patients also received between 6 weeks and 3 months of antibiotics according to the susceptibility of their pathogen(s). | All patients were successfully treated with no recurrence of initial infection at follow-up appointments of 8-16 months. | 4 | 0 | 0 | ‘Bacteriophage administration via the described route was generally well-tolerated, although one patient developed local redness and experienced pain during the rinsing procedure after seven days of treatment with the Pyo bacteriophage preparation. These symptoms were attributed to stowing, and subsided after phage therapy was stopped. However, a local immune reaction could not be ruled out because Pyo bacteriophage is not free from endotoxins.’  ‘No severe systemic  side effects or immune reactions were noted. In all patients, the systemic inflammatory markers (CRP  and WBC count) decreased to normal levels after one month, and no antibodies were produced against  the administered phages.’ |
| Tkhilaishvili *et al.* (2020), [38]  Germany  Case report | 1/1  *Pseudomonas aeruginosa* (two isolates: one sensitive only to colistin, one sensitive only to colistin and ceftazidime),  *Staphylococcus haemolyticus*,  *Staphylococcus epidermidis*. | An 80-year-old female with metabolic syndrome had a relapsing right knee prosthetic joint infection and chronic osteomyelitis of the femur secondary to a gunshot wound. This episode of infection was detected three months after re-implantation. | Sensitivity confirmed. | Anti-Pseudomonas phage at 10^9^ PFU/ml (Eliava Institute, Tbilisi). | During surgery the wound was rinsed with 2-3% sodium bicarbonate and an antibiotic-loaded cement spacer inserted. A 100ml intraoperative dose of phage was given. Thereafter, 5ml of phage at 10^8^ PFU/ml was administered via each of four wound drains every 8h for 5 days. Intravenous meropenem (1g/12h), colistin (150mg/24h) and ceftazidime (2g/12) were also given. | The patient was culture negative for Pseudomonas upon presentation with a subsequent *Staphylococcus epidermidis* infection 2 weeks later. Ten months after phage therapy the patient had no pain in the right knee, satisfactory mobility and no remarkable features at the surgical site. | 1 | 0 | 0 | ‘The combined antibiotic/phage treatment eradicated the infection and no side effects to phages were observed.’ |
| Doub *et al.* (2020), [32]  USA  Case report | 1/1  Methicillin resistant *Staphylococcus aureus.* | A 72-year old morbidly obese male with an MRSA prosthetic joint infection of the right knee. The infection was refractory to intravenous daptomycin, intravenous and intra-articular vancomycin and doxycycline. | Sensitivity confirmed. | Anti-staphylococcal phage SaGR51φ1 (Adaptive Phage Therapeutics, US). | After DAIR the patient was given two doses of intra-articular phage (5.4 x 10^9^ PFU) in 10ml of saline and was started on daptomycin (1g/day, 6 weeks). Daily intravenous phage (2.7 x 10^9^ PFU in 50ml of saline) was started the next day. At week 9 post-DAIR the patient underwent a further DAIR procedure and despite no clinical manifestation of infection another intraarticular administration (as before) of phage. Post-op cultures were negative. A further intra-articular administration was given two months later when the patient underwent further orthopaedic surgery. Post-op cultures were negative. | Treatment was successful with all subsequent surgical site cultures, taken at 9 and 17 weeks after the initial DAIR procedure, culture negative. | 1 | 0 | 0 | After the third intravenous dose of phage the patient developed raised AST and ALT. Phage was stopped after the third dose, daptomycin was continued. The patient’s AST and ALT returned to normal 10 days later. The authors proposed that the patient’s pre-existing hepatomegaly (? non-alcoholic fatty liver disease) meant that when the liver was challenged to clear substantial numbers of phage from the bloodstream local inflammatory changes occurred, causing transaminitis. This is the first report documenting this and the authors suggest cautionary, monitored, use of intravenous phage in patients with pre-existing liver pathology. |
| Cano et al.  (2020), [27]  USA  Case report | 1/1  *Klebsiella pneumoniae.* | A 62-year-old male with obesity, diabetes and a history of multiple right knee prosthetic joint infections. His *Klebsiella* infection was refractory to suppressive minocycline. | Sensitivity confirmed. | A novel naturally occurring phage (KpJH46φ; Adaptive Phage Therapeutics, US). | The patient received 40 daily intravenous infusions of 50ml of 6.3 x 10^10^ phages in 0.9% saline. Minocycline (200mg/day) was continued. | Erythema improved rapidly after two phage administrations and pain decreased until none remained at the end of therapy. Resolution was maintained at follow-up 34 weeks after therapy. | 1 | 0 | 0 | ‘The patient did not experience treatment-related adverse effects.’ There was no change in anti-phage antibodies over time, suggesting no immune response to phage.  ‘Reports such as this  one suggest that phage therapy may be safe, effective, and well  tolerated.’ |
| Ferry et al.  (2020), [26]  France  Case report | 1/1  Methicillin susceptible *Staphylococcus aureus*. | A 49-year-old male with relapsing  *S. aureus* knee megaprosthesis infection, resistant only to penicillin. | Sensitivity confirmed. | Two anti-staphylococcal phages, PP1493 and PP1815, were provided at 10^10^ PFU/ml suspended in Dulbecco’s phosphate buffered saline (Pherecydes Pharma, France). The phages were mixed with sterile water and a commercially available hydrogel for clinical use. | The phage-containing hydrogel was applied to the surface of the megaprosthesis during a debridement, antibiotics and implant retention (‘DAIR’) procedure. Empirical daptomycin (850mg/day) and tigecyline (100mg loading dose, and 50mg every 12h thereafter) were started post-operatively. | Five days after surgery the local surgical site appeared favourable, but the patient suffered a myocardial infarction. Anticoagulant therapy led to a haematoma at the surgical site which was found to be colonised with multiple bacterial species, none of which were *S. aureus*. Further antibiotic and surgical therapy were unable to restore the surgical site and the patient required transfemoral amputation approximately 1 year after receiving phage therapy. Surgical cultures from the amputation showed multiple bacterial species but were not positive for the *S. aureus* targeted by phage therapy. | 0 | 1 | 0 | The myocardial infarction and subsequent haematoma-related complications were not considered to be related to phage therapy. |
