# Supplementary material for: The Safety and Efficacy of Phage Therapy for Bone and Joint Infections: A Systematic Review
Source: Antibiotics (Basel). 2020 Nov 10;9(11):795. doi: 10.3390/antibiotics9110795 (PMC7697170; doi:10.3390/antibiotics9110795)
Supplement: Supplementary file 1 [file antibiotics-09-00795-s001.zip › Supplementary File S3.docx]

**Supplementary file three: critical appraisal**

*Critical appraisal of manuscripts was performed using the Joanna Briggs Institute suite of critical appraisal tools available from* <https://joannabriggs.org/critical-appraisal-tools> [accessed 14/09/2020]. The case series tool was adapted to include comments on adverse effects and, where appropriate, examples of what each question was interrogating.

**Case series**

| Author  (year),  [citation] | Clear inclusion criteria? | Reliable standard measurement of presenting condition? | Valid identification of infection (e.g. defined microbiology)? | Consecutive inclusion? | Complete inclusion and reporting? | Clear patient demographic data (e.g. range and mean of patient age)? | Clear clinical reporting (pre- treatment)? | Clear outcome or follow up reporting (e.g. clinical outcome)? | Comments on adverse effects? | Clear site demographic (e.g. study location)? | Appropriate statistical analysis, if present? | Comments |
| --- | --- | --- | --- | --- | --- | --- | --- | --- | --- | --- | --- | --- |
| Albee (1933), [41] | Yes | No | No | Yes | Unclear | No | No | No | No | No | N/A |  |
| Bernstein (1940), [24] | No | Unclear | No | Unclear | Yes | No | No | Yes | Yes | No | N/A |  |
| Baker (1963), [20] | Yes | Yes | Yes | No | No* | Yes | Yes | Yes | Yes | No | N/A | *States the ‘above case reports are a few of many which give evidence to the value and safety of SPL’. |
| Matusis (1974), [25] | Yes | Unclear | Yes | Unclear | No* | No | No | Yes | No | Yes | N/A | *65/89 cases included. The report states 94 patients but data for 5 are absent. |
| Slopek *et al.* (1987), [19] | No | Yes | Yes | Unclear | Yes | Yes | Yes | Yes | Yes* | Yes | Yes | *See preliminary paper Slopek *et al.* 1983 [42] |
| Southwest Regional Woundcare Centre (2006), [23] | No | Unclear | No | No | Unclear | Yes | Yes | Yes | No | Yes | N/A |  |
| Fish *et al.* (2016), [33] | Yes | Yes | Yes | Unclear | No* | Yes | Yes | Yes | Yes | Yes | N/A | *Nine patients treated; six ‘representative’ cases presented. |
| Fish *et al.* (2018), [35] | Yes | Yes | Yes | Unclear | Yes | Yes | Yes | Yes | Yes | No | N/A |  |
| Onsea *et al.* (2019), [37] | Yes | Yes | Yes | Unclear | Yes | No | Yes | Yes | Yes | Yes | N/A |  |

**Case reports**

| Author  (year)  [citation] | Were the patient’s demographic characteristics clearly reported? | Was the patient’s history clearly described? | Was the current clinical condition clearly described? | Were diagnostic tests or methods and results clearly described? | Were the treatment(s) or intervention(s) clearly described? | Was the post-intervention clinical condition clearly described? | Were any adverse or unanticipated events clearly described? |
| --- | --- | --- | --- | --- | --- | --- | --- |
| Ferry *et al.* (2018), [39] | Yes | Yes | Yes | Yes | Yes | Yes | Yes |
| Ferry *et al.* (2018), [40] | Yes | Yes | Yes | Yes | Yes | Yes | Yes |
| Fish *et al.* (2018), [34] | Yes | Yes | Yes | Yes | Yes | Yes | No |
| Nir-Paz *et al.* (2019), [36] | Yes | Yes | Yes | Yes | Yes | Yes | Yes |
| Tkhilaishvili *et al.* (2020), [38] | Yes | Yes | Yes | Yes | Yes | Yes | Yes |
| Doub *et al.* (2020), [32] | Yes | Yes | Yes | Yes | Yes | Yes | Yes |
| Cano *et al.* (2020), [27] | Yes | Yes | Yes | Yes | Yes | Yes | Yes |
| Ferry *et al.* (2020), [26] | Yes | Yes | Yes | Yes | Yes | Yes | Yes |
